# Risk factors for subsequent suicidal acts among 12–25-year-old high-risk callers to a suicide prevention hotline in China: a longitudinal study

**DOI:** 10.1186/s13034-024-00765-5

**Published:** 2024-06-19

**Authors:** Jianlan Wu, Ruoyun Zhang, Liting Zhao, Yi Yin, Jing Min, Yiming Ge, Yang Luo, Peiyao Li, Lingling Li, Yongsheng Tong

**Affiliations:** 1 Peking University Huilongguan Clinical Medical School, Beijing, China 7 Nan Dian Road, Changping, 100096; 2 Beijing Suicide Research and Prevention Center, Beijing Hui Long Guan Hospital, Beijing, China; 3 WHO Collaborating Center for Research and Training in Suicide Prevention, Beijing, China

**Keywords:** Suicide risk, Adolescent, Young adult, Hotline, Longitudinal study, China

## Abstract

**Background:**

A few previous cross-sectional studies investigated correlated factors of suicidal ideation or suicide attempts among suicide prevention hotline callers; however, scarcely any evidence was from a longitudinal study. In addition, it is still unclear whether improvements in some suicide risk factors could reduce the occurrence of subsequent suicidal acts. This longitudinal study focusing on the risk factors for subsequent suicidal acts among adolescent and young adult callers with high suicide risk aims to fill this gap.

**Methods:**

This study recruited 12–25-year-old high-risk callers to a China nationwide suicide prevention hotline. Potential risk factors, including hopefulness, psychological distress, depression, history of suicide attempts, alcohol or substance misuse, and acute life events, were examined during the index calls, and improvements in hopefulness, psychological distress, and suicide intent were assessed before ending the index calls. The recruited callers were followed up 12 months after their index calls. The primary outcome was the occurrence of suicidal acts (suicide attempts or suicide death) during follow-up. Kaplan–Meier survival curves, log-rank tests, and Cox proportional hazards model were used.

**Results:**

During the follow-up period, 271 of 1656 high-risk adolescent and young adult callers attempted suicide, and seven callers died by suicide. After adjusting for demographic variables, low hopefulness (Hazard Ratio [HR] = 2.03, 95% Confidence Interval [CI]=[1.47, 2.80]) at the beginning of the index call was associated with a higher risk for subsequent suicidal acts, whereas improvements in psychological distress (HR = 0.61, 95%CI [0.41, 0.89]) and suicidal intent (HR = 0.56, 95%CI [0.38, 0.84]) during the index call reduced the risk of subsequent suicidal acts. In addition, alcohol or substance misuse (Model 2, HR = 1.65, 95%CI [1.11, 2.46]) and suicide attempt history(Model 1: one episode, HR = 1.96, 95%CI=[1.05, 3.66]; two or more episodes, HR = 2.81, 95%CI [1.59, 4.96]. Model 2: one episode, HR = 2.26, 95%CI [1.06, 4.82]; two or more episodes: HR = 3.28, 95%CI [1.63, 6.60]) were risk factors for subsequent suicidal acts.

**Conclusions:**

While suicide prevention hotline operators deliver brief psychological interventions to high-risk adolescent and young adult callers, priority should be given to callers with low hopefulness and to the alleviation of callers’ high psychological distress and suicide intent.

**Supplementary Information:**

The online version contains supplementary material available at 10.1186/s13034-024-00765-5.

## Introduction

Adolescents and young adults (AYA), including individuals aged 12–25, undergo a pivotal phase of cognitive, emotional, and social skill maturation [1, 2]. Suicide among AYA is a major global public health concern globally [3, 4]. In many countries, suicide is the second or third leading cause of mortality among AYA [5, 6]. According to the Chinese Health Statistical Yearbook (2020), although suicide rates among AYA have shown a declining trend, suicide persistently ranks as the third leading cause of non-disease-related mortality in this age group in China [7].

Hotlines provide anonymous, convenient, remotely accessible, and free support to callers encountering psychological disturbances [8, 9], which aligns with the World Health Organisation’s advocacy for accessible mental health services [10]. Hotlines have become essential for AYA to seek psychological support [11, 12]. In China, half of the callers who sought help via hotlines struggled with suicide-related psychological disturbances, and such disturbances were more common in AYA than in all-age group callers [13–15]. Psychological interventions via hotlines were generally effective in reducing feelings of hopelessness, lowering immediate suicide intent, and effectively reducing psychological distress [16–19].

Previous studies have revealed that many factors, including conflicts with parents, deteriorated family relationships, lack of social support, childhood adversity, and history of suicide attempts, are associated with suicidal ideation and acts among AYA [4, 20–23]. However, few studies have focused on AYA seeking help from suicide prevention hotlines. Most existing studies were cross-sectional and investigated the correlations between suicidal ideation and the aforementioned factors. For instance, a study in Japan found a significant correlation between suicidal ideation and familial issues, such as abuse, family dissolution, and domestic violence [24]. A study on a hotline in China found that independent risk factors for suicidal ideation among young callers were being female and younger and having acute or chronic life events, a history of suicide attempts, high psychological distress, and severe depression [25]. Another study examined the risk factors for suicide attempts in the last two weeks among hotline callers of all age groups and reported similar results [26].

Risk factors for suicidal acts among high-risk AYA callers remain unclear, considering the distinct characteristics of callers at high versus low risk for suicide [27] and differences in characteristics between AYA and other age groups among high-risk hotline callers [14, 15]. Thus, the risk factors for suicidal acts, including suicide attempts and death by suicide, among high-risk AYA hotline callers must be identified using a longitudinal study design. Previously identified suicide risk factors could be classified as unmodifiable and modifiable factors. The unmodifiable factors refer to unchanged factors, including race, age, sex, etc.; modifiable factors refer to many social-psychological characteristics that could be improved by deliberate intervention. This study emphasised whether the elimination or alleviation of modifiable risk factors, such as low hopefulness, high psychological distress, and suicidal intent, reduces the risk of subsequent suicidal acts.

The Beijing Psychological Support Hotline regularly collects information on received calls and follows up with all callers assessed as high-risk 12 months after the call. This study extracted information from the hotline database and sought to provide evidence of risk factors for suicidal acts among AYA callers and suggestions for preventing suicidal acts in this population.

## Methods

### Study setting, design, and participants

This longitudinal study investigated callers to the Beijing Psychological Support Hotline, established in 2002. Although the hotline is located in Beijing, it serves callers across China [14]. The hotline is one of the largest crisis lines for suicide prevention in China and delivers free 24/7 psychological services nationwide. It receives 20,000–30,000 calls annually. All callers identified as high-risk are promptly administered a semi-structured psychological intervention lasting up to 90 min and are followed up for up to 12 months, which aims to decrease the caller’s risk for suicide [18]. In follow-up calls, if the callers presented high suicide risk, psychological interventions including a safety plan would be delivered by hotline counsellors. Details of the intervention were described in a previous study [18].

From January 2017 to December 2018, AYA callers assessed as being high suicide risk were recruited. High suicide risk was defined as meeting any one of the three conditions: (1) having a suicide plan and will make suicidal acts within the next 72 h, (2) reporting a suicide attempt in the past two weeks, and (3) ongoing suicidal acts several minutes before or during the call. The participants were followed up with 12 months after the call. The inclusion criteria were (1) being 12–25 years old at the index call (the first call included during the study period), (2) the index call being answered between 1 January 2017 and 31 December 2018, and (3) meeting the criteria of high suicide risk. The exclusion criteria were (1) a lack of baseline assessment for any reason and (2) refusal to follow-up at the end of the index call.

### Sample size calculation

The primary outcome of this study was subsequent suicidal act including suicide death or suicide attempt. The assumed primary risk factor was low hopefulness at the beginning of the index call. Previous studies have indicated that approximately 10% of high-risk AYA callers exhibit subsequent suicidal acts [18, 21]. The estimated relative risk of low hopefulness for subsequent suicidal acts was 2 [26]. A sample size estimation formula for cohort studies was used [29]. Considering a power of 0.9, *α* = 0.05, and a two-sided test, the required sample size was 494. Considering the potential loss of 20% of participants during follow-up, the expected sample size was 593.

### Data collection and measurement

Baseline and follow-up data were collected by qualified psychological counsellors who served as operators at the Beijing Psychological Support Hotline. The counsellors underwent rigorous and systematic training provided by the hotline [30].

The hotline’s “call-on” system [18] was developed to assist operators with taking calls and data collection and storage. While receiving a call, questions or prompts containing one or more blank spaces are automatically presented on a screen in front of the operator. The operator fills in the blanks by gathering information from the caller. The collected data is automatically saved in a predefined format. The baseline measurement included several factors, such as demographic characteristics, hopefulness, psychological distress, suicide intent, depression, suicide attempt history, relatives or acquaintances’ history of suicidal acts, alcohol or substance misuse, acute life events, chronic life events, history of being abused, fear of being attacked, and physical illness.

To assess hopefulness, psychological distress, and suicide intent, callers were asked to rate their experiences on a scale of 0 to 100 at the beginning and end of the index call. A score of 0 indicated no hope, distress, or suicide intent, whereas a score of 100 represented the maximum level of hope, extreme distress, or suicide intent. To assess depression, a structured depression screening questionnaire was adapted from a previous study on suicidality for use on hotlines [31]. The questionnaire evaluated the caller’s depressive symptoms and duration. The system automatically calculated a score to evaluate the caller’s depressive mood within the past two weeks. The total score ranged from 0 to 100, with higher scores indicating more severe depression. Relatives or acquaintances’ history of suicidal acts referred to whether any blood relatives, non-blood relatives, or friends of the caller had a history of suicide or suicide attempts. When enquiring about alcohol and substance misuse, operators asked callers if they had experienced repeated episodes of excessive alcohol consumption in the past year and if they had been using hypnotics, anti-anxiety drugs, anaesthetics, stimulants, or any drugs excessively, casually, or continuously for more than three months in the past year. Alcohol or substance misuse was determined if the answer to any of these questions was ‘yes’ and whether this had a moderate or significant impact on their daily life, social interactions, or workability in the past month. Callers’ experiences of chronic and acute life events, history of abuse, and severe physical illness were recorded if these factors had moderate, severe, or significant impacts. Fear of being attacked referred to callers frequently worrying about being attacked by others during the past month.

The short-term effects of the hotline intervention were assessed by measuring changes in hopefulness, psychological distress, and suicide intent during the index call. To calculate these changes, the scores reported by callers at the end of the call were subtracted from those reported at the beginning of the call. An increased score for hopefulness and decreased scores for psychological distress and suicide intent indicated improvements in these aspects.

The primary outcome was the occurrence of suicidal acts, including both suicide deaths and attempts, during the follow-up period. The occurrence of subsequent suicidal acts was assessed at each follow-up call. Information on death by suicide was obtained from the family members of deceased callers, and data on suicide attempts were provided by the callers themselves. Operators enquired whether the callers had any suicidal acts since the index call or last follow-up. In cases where callers could not be reached, family members who answered the follow-up call were asked if the caller had died by suicide.

Hotline operators informed high-risk callers of follow-up arrangements before ending the call. Operators clearly explained the purpose of the follow-up and informed them of the specific follow-up time to minimise the loss of follow-up. In cases where the caller could not be reached at the agreed-upon time, the operators were required to make three consecutive calls at different times on the same day. Callers were considered ‘lost’ after three unsuccessful calls, with the follow-up process continuing to the next scheduled follow-up. Follow-ups were conducted one day, one week, one month, three months, six months, and 12 months after the index call, totalling six follow-ups via telephone.

The study endpoint was defined as the date of the first suicidal behaviour, including suicide attempts and death by suicide. The censoring dates were determined based on the date of last contact and interview, death from causes other than suicide, or 12 months after the index call, whichever occurred first. The entry date was defined as the date of the index call.

### Statistical analysis

Continuous variables, such as hopefulness, psychological distress, suicide intent, and depression, were transformed into categorical variables based on their medians. Age was categorised into two groups: adolescents (aged 12–17) and young adults (aged 18–25).

Baseline characteristics (i.e. sex, age, hopefulness, psychological distress, and suicide intent) were compared between callers who did and did not follow up using *χ2* tests. Kaplan–Meier survival curves and log-rank tests were used to investigate the potential suicide risk factors collected in the baseline assessment. Survival analysis was conducted using the Cox proportional hazards model under the assumption of proportional hazards to identify risk factors associated with suicidal acts during follow-up. The variables incorporated into the Cox regression analysis were those that exhibited *p*-values less than 0.30 [32] in the log-rank tests. A stratified Cox regression analysis was performed if the data did not meet the proportional hazards assumption. *P*-values < 0.05 were considered statistically significant.

Two Cox regression models were used. This population exhibited a high collinearity between marital status, education level, and age. Therefore, in the Cox regression, we adjusted for age and gender, without including marital status and education, to ensure stability and reliability. In Model 1, we adjusted for sex and age; alcohol or substance misuse, acute life events, severe physical illness, severe depression, suicide attempt history, low hopefulness, and high suicide intent at the beginning of the index call were included and screened. In Model 2, we adjusted for gender and age; low hopefulness and high suicide intent at the beginning of the index call were replaced with improvements in hopefulness, psychological distress, and suicide intent. The remaining variables in Model 1 were retained and screened in Model 2. The callers were de-identified before data analysis. Data analyses were performed using SPSS 18.0.

## Results

**Fig. 1:**
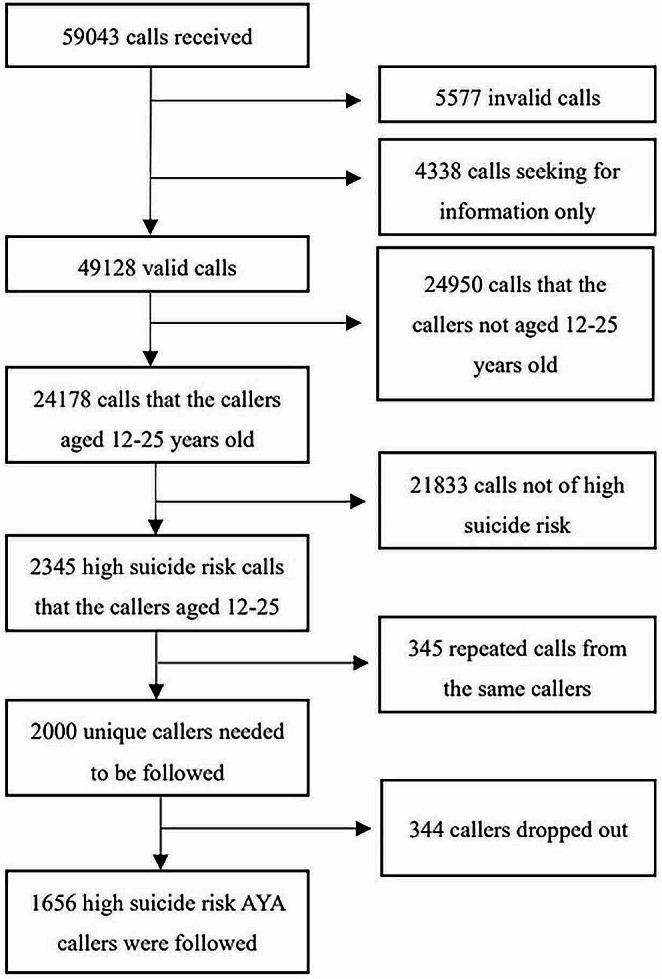
Flowchart of screening participants met for inclusion and exclusion criteria

The details of the recruitment and follow-up processes are shown in Fig. 1. In summary, 2345 high-risk calls that met the age criteria and were assessed as high suicide risk were included. Among them, 345 were repeat calls from the same callers, resulting in a cohort of 2000 high-risk callers for this study.

During the 12-month follow-up, 344 callers (17.2%) could not be contacted. There was no statistically significant difference between followed and non-followed callers in terms of sex (*χ2* = 0.29, *p* = 0.589) or age (adolescent or young adult, *χ2* = 2.84, *p* = 0.092). Statistically significant differences were found between the two groups of callers in terms of education level (*χ2* = 18.03, *p* < 0.001), history of being abused (*χ2* = 7.31, *p* = 0.007), and high suicide intent at the beginning of the index call (*χ2* = 6.06, *p* = 0.014). The non-followed callers were more likely to report high suicide intent than the followed callers. Details of the comparisons between the followed and non-followed groups are shown in Supplemental Table 1.

The study followed up at least once with 1656 participants (82.8%), comprising 650 males and 1006 females. 1656 participants were followed up for a cumulative total of 8045 person-months. Within this cohort, 278 individuals (16.8% of the followed-up callers) enacted suicidal acts during the 12 month period after the index call. Among them, seven died by suicide (3 males and 4 females), and the other 271 attempted suicide. Among the 271 callers with suicide attempts, 264 reported 427 suicide attempt episodes, and the other seven did not report the precise number. Six of the seven suicides occurred within 1 month, and all suicides were among young adults. In addition, 60.1% (163/271) of the first suicide attempts occurred within one month after the index call, and 74.5% (202/271) of the first episodes occurred within three months.

Table 1 presents the baseline characteristics of the callers, including those with and without suicidal acts during the follow-up period. No statistically significant differences were observed between callers with and without suicidal acts based on sex (*χ2* = 1.02, *p* = 0.313). Young adult callers were less likely than adolescent callers to enact suicidal acts during the follow-up period (*χ2* = 9.62, *p* = 0.002). Furthermore, callers who reported low hopefulness or high suicide intent at the beginning of the index call and callers with alcohol or substance misuse or a history of suicide attempts were more likely to report suicidal acts during the follow-up period. In addition, the short-term effects of the hotline interventions were examined. Callers who reported improvement in any of the three aspects (i.e. hopefulness, psychological distress, or suicide intent were less likely to enact suicidal acts during the follow-up period (Table 1).

**Table 1:** Comparisons of baseline characteristics between callers with and without subsequent suicidal acts

Variables	Without subsequent suicidal acts ^a^		With subsequent suicidal act ^b^		Log rank	*p* value
	(*n* = 1378)		(*n* = 278)			
	n	%	n	%	χ2	
Male	549	39.8	101	36.3	1.02	0.313
Age						
Adolescents (12–17 years old)	429	31.1	100	36.0	9.62	**0.002**
Young adults (18–25 years old)	949	68.9	178	64.0		
Education						
Elementary school and below	30	2.18	6	2.16	6.54	0.088
Middle school	293	21.3	67	24.1		
High school	507	36.8	92	33.1		
University and above	532	38.6	108	38.8		
Marital status						
Never married	1326	96.2	271	97.5	1.04	0.309
Ever married or co-habiting	49	3.56	7	2.52		
Suicide plan						
Will conduct suicidal acts immediately or in 72 h	595	43.2	111	39.9	0.77	0.379
An ongoing suicidal act, or attempted suicide in last 2 weeks	783	56.8	167	60.1		
Alcohol or substance misuse	146	10.6	41	14.7	7.38	**0.007**
Chronic life events	727	52.8	145	52.2	1.05	0.305
Acute life events	689	50.0	121	43.5	1.18	0.277
Severe physical illness	131	9.51	32	11.5	2.82	0.093
History of being abused	246	17.9	47	16.9	0.01	0.918
Fear of being attacked	298	21.6	58	20.9	0.35	0.557
Relatives or acquaintances suicidal acts history	564	40.9	113	40.6	0.05	0.821
Low hopefulness at the beginning of index call (score 0–10)	672	48.8	159	57.2	19.93	**< 0.001**
High psychological distress at the beginning of index call (score 90–100)	691	50.1	141	50.7	0.506	0.477
High suicide intent at the beginning of index call (score 80–100)	713	51.7	171	61.5	10.78	**< 0.001**
Severe depression (score 77–100)	515	37.4	107	38.5	2.01	0.156
Improvement in hopefulness ^c^	391	28.4	56	20.1	5.36	**0.021**
Improvement in psychological distress ^d^	715	51.9	111	39.9	18.39	**< 0.001**
Improvement in suicide intent ^e^	739	53.6	122	43.9	12.08	**0.001**
Suicide attempt history						
0	195	14.2	18	6.47	17.79	**< 0.001**
One episode	250	18.1	39	14.0		
Two or more episodes	645	46.8	147	52.9		

Bold values indicate statistical significance

^a^Without subsequent suicidal acts refers to AYA callers at high suicidal risk who did not report suicidal acts during 12-month follow-up. The total sample sizes for some variables were not 1378 because of data missing

^b^With subsequent suicidal acts refers to AYA callers with high suicidal risk who reported suicide or suicide attempt during 12-month follow-up. The total sample sizes for some variables were not 278 because of data missing

^c^Improvement in hopefulness means: hopefulness at the end of the index call - hopefulness at the beginning of the index call > 0

^d^Improvement in psychological distress means: psychological distress at the end of the index call - psychological distress at the beginning of the index call < 0

^e^Improvement in suicide intent means: suicide intent at the end of the index call - suicide intent at the beginning of the index call < 0

**Fig. 2:**
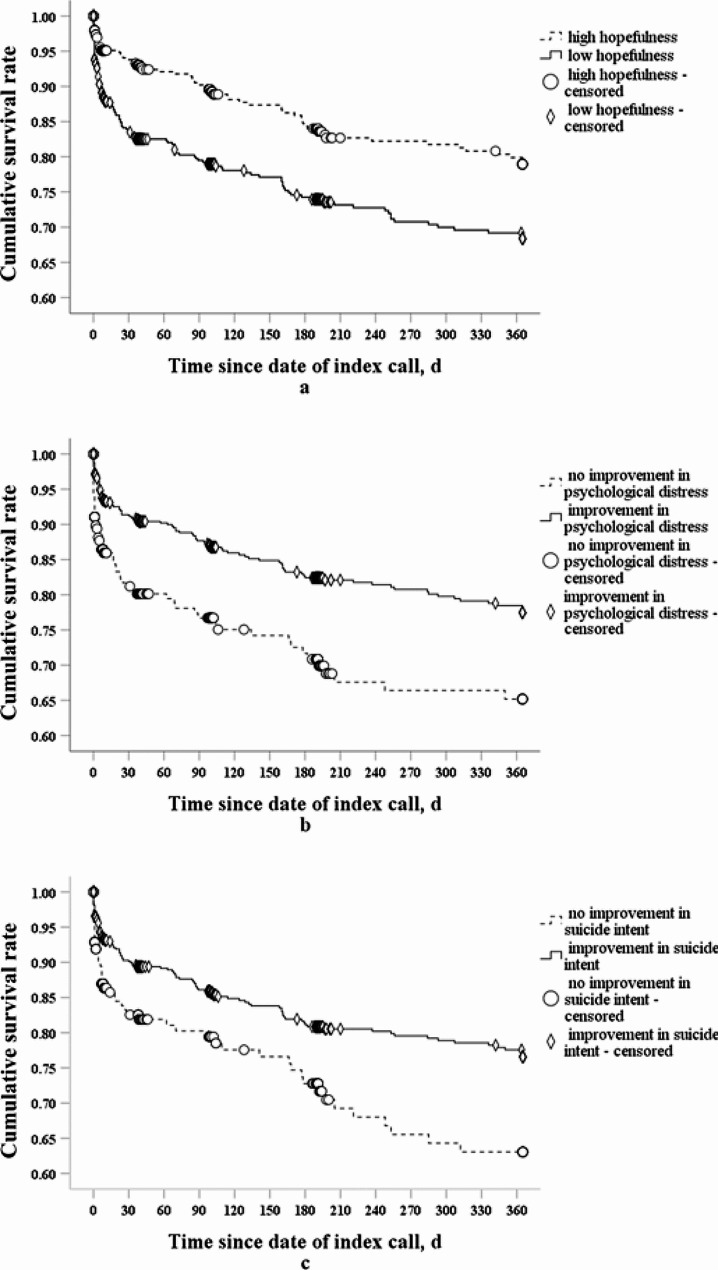
Survival rates of high-risk AYA callers with certain factors using Kaplan-Meier survival curves. **a**: Cumulative survival rate of high-risk AYA callers among those with high hopefulness and those with low hopefulness at the beginning of index call. **b**: Cumulative survival rate of high-risk AYA callers among those who reported improvement in psychological distress and those who did not.** c**: Cumulative survival rate of high-risk AYA callers among those who reported improvement in suicide intent and those who did not

As shown in Fig. 2, compared to callers with high hopefulness at the beginning of the index call, those with low hopefulness were more likely to make suicide acts during the follow-up period. Additionally, callers who reported improvements in psychological distress or suicide intent were less likely to make suicide acts during the follow-up period, compared with those who did not.

Table 2 presents the risk factors for suicidal acts during the follow-up period. Young adults were less likely than adolescents to enact suicidal acts (Model 1: Hazard Ratio [HR] = 0.70; Model 2: HR = 0.66). After demographic variables were adjusted, alcohol or substance misuse (Model 2: HR = 1.65) and suicide attempt history (Model 1: one episode, HR = 1.96; two or more episodes, HR = 2.81. Model 2: one episode, HR = 2.26; two or more episodes, HR = 3.28) were associated with a higher risk of subsequent suicidal acts. In Model 1, low hopefulness at the beginning of the index call (HR = 2.03) was identified as a risk factor for suicidal acts during the follow-up period. In Model 2, improvements in psychological distress (HR = 0.61) and suicide intent (HR = 0.56) were associated with a lower risk of subsequent suicidal acts.

**Table 2:** Correlates of baseline characteristics and suicidal acts during follow up period

Variables	Model 1^a^		Model 2^b^	
	HR	95% CI	HR	95% CI
Male	0.99	0.72–1.37	1.00	0.69–1.45
Age				
Adolescents	1		1	
Young adults	**0.70**	0.51–0.95	**0.66**	0.46–0.93
Alcohol or substance misuse	–		**1.65**	1.11–2.46
Suicide attempt history				
0	1		1	
One episode	**1.96**	1.05–3.66	**2.26**	1.06–4.82
Two or more episodes	**2.81**	1.59–4.96	**3.28**	1.63–6.60
Improvement in suicide intent	–		**0.56**	0.38–0.84
Improvement in psychological distress	–		**0.61**	0.41–0.89
Low hopefulness at the beginning of index call (score 0–10)	**2.03**	1.47–2.80	–	

Bold values indicate statistical significance

*HR * hazard ratio, *CI *confidence interval

^a^ Model 1: sex and age of callers were adjusted, and alcohol or substance misuse, acute life events, severe physical illness, severe depression, suicide attempt history, low hopefulness at the beginning of the index call and high suicide intent at the beginning of the index call were included and screened

^b^ Model 2: sex and age of callers were adjusted, and low hopefulness and high suicide intent at the beginning of the index call were replaced with improvements in hopefulness, psychological stress, and suicide intent. The remaining variables from Model 1 were retained and screened in Model 2

## Discussion

### Main findings

This study examined 1656 AYA callers to a nationwide suicide prevention hotline in China who were at high risk for suicide. This study explored the risk factors associated with suicidal acts over a 12 month follow-up period. In addition to alcohol or substance misuse and suicide attempt history, several aspects of subjective experience, such as low hopefulness at the beginning of the index call and short-term improvement in psychological distress and suicide intent, were associated with the occurrence of suicidal acts during the follow-up period. These findings indicated that, in addition to unmodifiable risk factors, modifiable risk factors, such as hopefulness, psychological distress, and suicide intent, were associated with the risk of subsequent suicidal acts among high-risk AYA callers. Furthermore, short-term improvements in these modifiable factors could substantially reduce the risk of subsequent suicidal acts.

In our study, 16.8% (278) of the participants reported subsequent suicidal acts during the follow-up period, whereas 0.4% (7) died by suicide. This rate is substantially higher than that observed in high-risk hotline callers of all age groups [18] and other general adolescent and young adult cohorts [4, 7]. Due to insufficient awareness of mental health issues, stigma associated with suicide, and concerns about the financial burden of treatment, AYA are significantly less likely to seek help from psychiatric institutions [33–35]. They are more likely to turn to psychological support hotlines to alleviate their psychological distress [14, 15]. This emphasises that psychological support hotlines should be one of the main measures for suicide prevention among AYA.

Suicide risk was higher in the first three months after the index call. More than 70% of suicide attempts and six of seven deaths by suicide occurred within 90 days. This aligns with previous findings [18], underscoring the need to focus on high-risk callers within the first three months following an index call. AYA callers require particular attention in the first three months after a high-risk call, including informing their family members and friends to construct safety plans and, when necessary, refer them to mental health professionals or institutions.

Low hopefulness was a significant risk factor for suicidal acts, which was in line with previous studies [26]. However, only 27% (447/1656) of the participants demonstrated improvements in hopefulness. In addition, the Cox regression analysis did not show a statistically significant correlation between improvement in hopefulness and the risk of subsequent suicidal acts. Individuals experiencing suicidal ideations often perceive their problems and negative emotions as unchangeable, endless, and unbearable [36, 44, 45]. Enhancing hopefulness through one telephone intervention that focuses on emotional relief is challenging.

Approximately half of the participants showed improvements in psychological distress (826/1656) and suicide intent (861/1656) after the short-term intervention delivered by the hotline. This indicates that short-term interventions could be effective in alleviating callers’ psychological distress and suicide intent, which is consistent with previous research findings [18]. Improvements in psychological distress and suicide intent substantially reduced the risk of subsequent suicidal acts among high-risk AYA callers. The main purpose of the hotline intervention is to focus on immediate suicidal risk, ensure the safety of individuals, and provide immediate emotional relief and reassurance [11]. This may have contributed to the alleviation of callers’ psychological distress and suicidal intent during and after the index call. Our findings suggest that, although the psychological help delivered by the hotline was a short-term intervention, its effectiveness lasted long-term, and improvements in psychological distress and suicide intent were strongly associated with the reduction in subsequent suicidal acts. Methods of eliminating or alleviating these two modifiable factors should be identified when developing hotline-based intervention strategies for AYA callers at high suicide risk.

Young adults, as compared to adolescents, were found to be less likely to make suicidal acts in this study. Previous studies yielded different results in this regard [7, 36, 37]. Adverse life events in schools, families, and other sources can lead to mental health issues among adolescents and young adults [38, 39]. In China, compared to individuals aged 12–17, those aged 18–25 may face less academic pressure and fewer negative family environments; however, they may encounter more issues, such as romantic relationship problems. The hotline in our study was designed to provide structured consultation services to all-age populations; therefore, assessments of issues unique to adolescent and young adult callers, such as academic difficulties, school bullying, and absenteeism, remain insufficient. Further research is required to explore the differences in suicide rates and unique influencing factors between the two age groups.

Severe depression evaluated at the index call did not emerge as a significant risk factor in this longitudinal study, which diverges from previous research findings [40, 41]. Previous studies indicated that depression is a significant risk factor for suicidal acts [42, 43]. Potentially significant fluctuations in depressive mood after baseline assessment might have resulted in a weakened association with suicidal acts during follow-up. Therefore, future research should reevaluate the level of depression during follow-up.

## Strengths and limitations

This study had several strengths. First, this longitudinal study had a follow-up period of 12 months. Second, the primary outcome was the occurrence of suicide or suicide attempts, which is a more direct and clinically significant measure compared to previous studies that focused on depressive symptoms or suicidal ideation. Third, the sample size was large, which enhanced the statistical power and generalisability of the findings. Finally, this study provided valuable insights into the long-term effects of short-term psychological interventions for high-risk hotline callers and shed light on the impact of these interventions on subsequent suicidal acts.

Nevertheless, this study had some limitations. First, the hotline service was primarily designed to offer structured consultations and interventions to the general population, which resulted in an insufficient assessment of issues specific to AYA. Future studies should incorporate a more targeted assessment of callers in the AYA group. Second, the reliance on self-reports from callers for factors such as acute life events and outcomes such as suicide attempts may have weakened the validity of the study. Third, the study sample only included callers assessed as having a high suicidal risk. Therefore, the findings of this study may not be generalisable to callers with moderate or low risk for suicide. Fourth, this study did not investigate the potential influence of factors that may have emerged during the follow-up period. Future studies should assess factors such as depression and recent acute life events at each follow-up assessment. Fifth, this study combined death by suicide and suicide attempts as primary outcomes due to the limited number of deaths observed. However, the risk factors associated with these two types of suicidal behaviours may differ. Sixth, about 20% high-risk AYA callers could not be contacted and followed, which limits the generalisability of our findings. Seventh, according to ethical requirements, all callers assessed as high suicide risk at the index call or follow-up phone call, had been intervened by hotline counsellors. This would impact the association between potential risk factors and subsequent suicidal acts. Finally, several potential confounding factors, such as psychiatric disorders, residential areas, and family socioeconomic status, were not assessed in this study. Further studies are required to address these limitations.

### Implication

Based on our findings, we propose several recommendations for hotlines delivering psychological interventions to AYA callers at high suicide risk. First, follow-up should be sustained for a minimum of three months. This extended follow-up period allows for ongoing monitoring and support, thus reducing the risk of further suicidal acts. Second, hotline interventions for high-risk AYA callers should focus on alleviating psychological distress and suicide intent. These interventions should be implemented during the follow-up period to provide continuous support.

## Conclusion

Our findings indicated that, in addition to unmodifiable factors, such as alcohol or substance misuse and suicide attempt history, modifiable factors, including low hopefulness, high suicide intent, and high psychological distress at the beginning of the index call, were associated with a reduction in subsequent suicidal acts among AYA callers at high suicide risk. A comprehensive assessment of the risk factors and high-quality psychological interventions targeting psychological distress and suicidal intent should be provided during hotline services for suicide prevention.

### Electronic supplementary material

Supplementary Material 1.

## Data Availability

No datasets were generated or analysed during the current study.

## Abbreviations

AYA: Adolescent and young adult
HR: Hazard ratio
CI: Confidential interval
GAE: Granulomatous amoebic encephalitis

## Declarations

### Abbreviations

AYA Adolescent and Young Adult.

HR Hazard Ratio.

CI Confidential Interval.

## References

[1] Patel PK, Leathem LD, Currin DL, Karlsgodt KH (2021). Adolescent neurodevelopment and vulnerability to psychosis. Biol Psychiatry.

[2] Luijten CC, van de Bongardt D, Jongerling J, Nieboer AP (2021). Longitudinal associations among adolescents’ internalizing problems, well-being, and the quality of their relationships with their mothers, fathers, and close friends. Soc Sci Med.

[3] World Health Organization (2020). Adolescent mental health.

[4] Liu XC, Chen H, Liu ZZ, Wang JY, Jia CX (2019). Prevalence of suicidal behaviour and associated factors in a large sample of Chinese adolescents. Epidemiol Psychiatr Sci.

[5] Heron M (2019). Deaths: leading causes for 2017. National Vital Statistics Reports.

[6] Kokkevi A, Rotsika V, Arapaki A, Richardson C (2012). Adolescents’ self-reported suicide attempts, self-harm thoughts and their correlates across 17 European countries. J Child Psychol Psychiatry.

[7] National Health Commission (2020). Chinese Health Statistical Yearbook.

[8] Coveney CM, Pollock K, Armstrong S, Moore J (2012). Callers’ experiences of contacting a national suicide prevention helpline: report of an online survey. Crisis.

[9] Hunt T, Wilson CJ, Caputi P, Woodward A, Wilson I (2017). Male suicide as a gendered phenomenon: implications for telephone crisis support. Aust N Z J Psychiatry.

[10] Action Mental Health Gap (2023). Programme (mhGAP) guideline for mental, neurological and substance use disorders.

[11] An J, Liu Z, Liang H, Tong YS, Huang YQ (2021). Psychological crisis intervention in public health emergencies. Chin Mental Health J.

[12] Gould MS, Lake AM, Galfalvy H, Kleinman M, Munfakh JL, Wright J (2018). Follow-up with callers to the national suicide prevention lifeline: evaluation of callers’ perceptions of care. Suicide Life Threat Behav.

[13] Kerner B, Carlson M, Eskin CK, Tseng CH, Ho JG, Zima B (2021). Trends in the utilization of a peer-supported youth hotline. Child Adolesc Ment Health.

[14] An J, Yin Y, Liang H, Zhao L, Tong Y (2023). Characteristics of adolescents who called the Beijing psychological support hotline. Chin Mental Health J.

[15] Zhao LT, Li CL, Wu MJ, Tong YS (2023). Comparisons of characteristics of adult and juvenile callers at high risk for suicide from psychological assistance hotline and the influencing factors of intervention effect. Sichuan Mental Health.

[16] Shaw FF, Chiang WH (2019). An evaluation of suicide prevention hotline results in Taiwan: caller profiles and the effect on emotional distress and suicide risk. J Affect Disord.

[17] Gould MS, Munfakh JLH, Kleinman M, Lake AM (2012). National suicide prevention lifeline: enhancing mental health care for suicidal individuals and other people in crisis. Suicide Life Threat Behav.

[18] Tong Y, Conner KR, Wang C, Yin Y, Zhao L, Wang Y (2020). Prospective study of association of characteristics of hotline psychological intervention in 778 high-risk callers with subsequent suicidal act. Aust N Z J Psychiatry.

[19] Mathieu SL, Uddin R, Brady M, Batchelor S, Ross V, Spence SH (2021). Systematic review: the state of Research Into Youth helplines. J Am Acad Child Adolesc Psychiatry.

[20] Wasserman D, Carli V, Iosue M, Javed A, Herrman H (2021). Suicide prevention in childhood and adolescence: a narrative review of current knowledge on risk and protective factors and effectiveness of interventions. Asia Pac Psychiatry.

[21] Liu RT, Walsh RFL, Sheehan AE, Cheek SM, Sanzari CM (2022). Prevalence and correlates of suicide and nonsuicidal self-injury in children: a systematic review and meta-analysis. JAMA Psychiatry.

[22] Björkenstam C, Kosidou K, Björkenstam E (2017). Childhood adversity and risk of suicide: cohort study of 548 721 adolescents and young adults in Sweden. BMJ.

[23] Siu AMH (2019). Self-harm and suicide among children and adolescents in Hong Kong: a review of prevalence, risk factors, and Prevention Strategies. J Adolesc Health.

[24] Ohtaki Y, Doki S, Kaneko H, Hirai Y, Oi Y, Sasahara S (2019). Relationship between suicidal ideation and family problems among young callers to the Japanese crisis hotline. PLoS ONE.

[25] An J, Yin Y, Liang H, Zhao L, Tong YS (2023). Risk factors of suicidal ideation among adolescents who called the Beijing psychological support hotline. Chin Mental Health J.

[26] Pang Y, Yang FD, Tong YS, Zhao LT, Wang CL, Liang H (2015). Related factors of attempted suicide among Beijing psychological aids hotline callers. Chin Mental Health J.

[27] Wang CL, Wang SL, Tong YS, Yang FD, Meng M (2011). Characteristics of high suicidal risk calls and effectiveness of crisis intervention by Beijing crisis hotline. Chin Mental Health J.

[28] Tong Y, Yin Y, Conner KR, Zhao L, Wang Y, Wang X (2023). Predictive value of suicidal risk assessment using data from China’s largest suicide prevention hotline. J Affect Disord.

[29] Huang YQ (2015). Control of random errors and determination of sample size in medical research. Chin Mental Health J 2015.

[30] Wang CL, Zhao XQ, Bueber M (2010). A year-long accreditation course for professional crisis hotline operators. China J Health Psychol.

[31] Phillips MR, Shen Q, Liu X, Pritzker S, Streiner D, Conner K (2007). Assessing depressive symptoms in persons who die of suicide in mainland China. J Affect Disord.

[32] Hosmer DW, Lemeshow S, Sturdivant RX (2013). Applied Logistic Regression.

[33] Tsamadou E, Voultsos P, Emmanouilidis A, Ampatzoglou G (2021). Perceived facilitators of and barriers to mental health treatment engagement among decision-making competent adolescents in Greece. BMC Psychiatry.

[34] Reardon T, Harvey K, Baranowska M, O’Brien D, Smith L, Creswell C (2017). What do parents perceive are the barriers and facilitators to accessing psychological treatment for mental health problems in children and adolescents? A systematic review of qualitative and quantitative studies. Eur Child Adolesc Psychiatry.

[35] Gulliver A, Griffiths KM, Christensen H (2010). Perceived barriers and facilitators to mental health help-seeking in young people: a systematic review. BMC Psychiatry.

[36] Fu X, Zhang K, Chen XF (2023). Report on National Mental Health in China (2021–2022).

[37] Miron O, Yu KH, Wilf-Miron R, Kohane IS (2019). Suicide rates among adolescents and young adults in the United States, 2000–2017. JAMA.

[38] Miller AB, Esposito-Smythers C, Weismoore JT, Renshaw KD (2013). The relation between child maltreatment and adolescent suicidal behavior: a systematic review and critical examination of the literature. Clin Child Fam Psychol Rev.

[39] Sacks V, Murphey D (2018). The prevalence of adverse childhood experiences, nationally, by state and by race or ethnicity. Child Trends Res Brief.

[40] Mojtabai R, Olfson M, Han B (2016). National trends in the prevalence and treatment of Depression in adolescents and Young adults. Pediatrics.

[41] Li F, Cui Y, Li Y, Guo L, Ke X, Liu J (2022). Prevalence of mental disorders in school children and adolescents in China: diagnostic data from detailed clinical assessments of 17,524 individuals. J Child Psychol Psychiatry.

[42] Bukstein OG (2022). Screening for adolescent depression and suicide risk. JAMA.

[43] Kalin NH (2021). Anxiety, depression, and suicide in Youth. Am J Psychiatry.

[44] Hawton K, Hill NTM, Gould M, John A, Lascelles K, Robinson J (2020). Clustering of suicides in children and adolescents. Lancet Child Adolesc Health.

[45] Hovey JD, Roley-Roberts ME, Hurtado G, Seligman LD, Levine JC, Kene P (2023). Coping competence and hopelessness moderate the influence of perceived burdensomeness on suicidal ideation in undergraduate college students. Curr Psychol.

