# Supplementary material for: Risk factors for subsequent suicidal acts among 12–25-year-old high-risk callers to a suicide prevention hotline in China: a longitudinal study
Source: Child Adolesc Psychiatry Ment Health. 2024 Jun 19;18:73. doi: 10.1186/s13034-024-00765-5 (PMC11188529; doi:10.1186/s13034-024-00765-5)
Supplement: Supplementary file 1 — Supplementary Material 1. [file 13034_2024_765_MOESM1_ESM.docx]

**Supplemental**

Table 1. Baseline characteristics of followed group and unfollowed group of high suicidal risk callers

| Variables | Followed group ^a^ | | Unfollowed group ^b^ | | χ2 | p value |
| --- | --- | --- | --- | --- | --- | --- |
|  | (n=1656) | | (n=344) | |  |  |
|  | n | % | n | % |  |  |
| Male | 650 | 39.3 | 140 | 40.7 | 0.29 | 0.589 |
| Age |  |  |  |  |  |  |
| Adolescents (12-17 years old) | 529 | 31.9 | 126 | 36.6 | 2.84 | 0.092 |
| Young adults (18-25 years old) | 1127 | 68.1 | 218 | 63.4 |  |  |
| Education level |  |  |  |  |  |  |
| Elementary school and below | 36 | 2.17 | 14 | 4.07 | 18.03 | **<0.001** |
| Middle school | 360 | 21.7 | 101 | 29.4 |  |  |
| High school | 599 | 36.2 | 111 | 32.3 |  |  |
| University and above | 640 | 38.6 | 104 | 30.2 |  |  |
| Marital status |  |  |  |  |  |  |
| Never married | 1597 | 96.4 | 330 | 95.9 | 0.02 | 0.880 |
| Ever married or co-habiting | 56 | 3.38 | 11 | 3.20 |  |  |
| Suicide plan |  |  |  |  |  |  |
| Will conduct suicidal acts immediately or in 72 h | 706 | 42.6 | 164 | 47.7 | 2.95 | 0.086 |
| An ongoing suicidal act, or attempted suicide in last two weeks | 950 | 57.4 | 180 | 52.3 |  |  |
| Alcohol or substance misuse | 187 | 11.3 | 39 | 11.3 | 3.15 | 0.076 |
| Chronic life events | 872 | 52.7 | 135 | 39.2 | 0.01 | 0.923 |
| Acute life events | 810 | 48.9 | 117 | 34.0 | 1.05 | 0.305 |
| Severe physical illness | 163 | 9.84 | 21 | 6.10 | 0.84 | 0.358 |
| History of being abused | 293 | 17.7 | 62 | 18.0 | 7.31 | **0.007** |
| Fear of being attacked | 356 | 21.5 | 65 | 18.9 | 2.60 | 0.107 |
| Relatives or acquaintances suicidal acts history | 677 | 40.9 | 104 | 30.2 | 0.03 | 0.863 |
| Low hopefulness at the beginning of index call  (score 0-10) | 831 | 50.2 | 167 | 48.5 | 1.46 | 0.227 |
| High psychological distress at the beginning of index call  (score 90-100) | 832 | 50.2 | 160 | 46.5 | 0.12 | 0.724 |
| High suicide intent at the beginning of index call  (score 80-100) | 884 | 53.4 | 199 | 57.8 | 6.06 | **0.014** |
| Severe depression  (score 77-100) | 622 | 37.6 | 106 | 30.8 | 0.78 | 0.378 |
| Improvement in hopefulness ^c^ | 447 | 27.0 | 56 | 16.3 | 0.76 | 0.385 |
| Improvement in psychological distress ^d^ | 826 | 49.9 | 109 | 31.7 | 1.23 | 0.267 |
| Improvement in suicide intent ^e^ | 861 | 52.0 | 114 | 33.1 | 2.57 | 0.109 |
| Suicide attempt history |  |  |  |  |  |  |
| 0 | 213 | 12.9 | 45 | 13.1 | 2.02 | 0.363 |
| One episode | 289 | 17.5 | 45 | 13.1 |  |  |
| Two or more episodes | 792 | 47.8 | 133 | 38.7 |  |  |

a: The total sample sizes for some variables were not 1656 because of data missing.

b: The total sample sizes for some variables were not 344 because of data missing.

c: Improvement in hopefulness means: hopefulness at the end of the index call - hopefulness at the beginning of the index call >0.

d: Improvement in psychological distress means: psychological distress at the end of the index call - psychological distress at the beginning of the index call <0.

e: Improvement in suicide intent means: suicide intent at the end of the index call - suicide intent at the beginning of the index call <0.
